# The Combining of Tyrosine Kinase Inhibitors and Immune Checkpoint Inhibitors as First-Line Treatment for Advanced Stage Hepatocellular Carcinoma

**DOI:** 10.3390/jcm11164874

**Published:** 2022-08-19

**Authors:** Shou-Wu Lee, Sheng-Shun Yang, Han-Chung Lien, Yen-Chun Peng, Chun-Fang Tung, Teng-Yu Lee

**Affiliations:** 1 Division of Gastroenterology, Department of Internal Medicine, Taichung Veterans General Hospital, Taichung 40705, Taiwan; 2 Department of Internal Medicine, Chung Shan Medical University, Taichung 40201, Taiwan; 3 Department of Internal Medicine, Yang Ming Chiao Tung University, Taipei 112304, Taiwan; 4 Department of Post-Baccalaureate Medicine, College of Medicine, Chung Hsing University, Taichung 40227, Taiwan; 5 Ph.D. Program in Translational Medicine, Chung Hsing University, Taichung 40227, Taiwan; 6 Institute of Biomedical Sciences, Chung Hsing University, Taichung 40227, Taiwan

**Keywords:** hepatocellular carcinoma, immune checkpoint inhibitor, tyrosine kinase inhibitor

## Abstract

Aim: Hepatocellular carcinoma (HCC) is one of the most common cancers. Tyrosine kinase inhibitors (TKIs), including sorafenib (SOR) and lenvatinib (LEN), as well as immune checkpoint inhibitors (ICIs), including nivolumab (NIVO) and pembrolizumab (PEMBRO), have been approved for the treatment of advanced HCC. The aim of the study is to determine whether advanced-stage HCC patients should receive a combination of TKI and ICI as first-line therapy. Methods: Data for subjects with BCLC stage C HCC, who were receiving combining TKI and ICI as first-line therapy at Taichung Veterans General Hospital from April 2019 to July 2021, were evaluated. The general and therapeutic outcome data were collected and analyzed. Results: A total of 33 patients were enrolled (8 SOR/NIVO, 4 SOR/PEMBRO, 11 LEN/NIVO, and 10 LEN/PEMBRO). All cases belonged to Child-Pugh class A. The objective response rate was 48.5%, and disease control rate was 72.7%. The average progression-free survival (PFS) and overall survival (OS) of all patients was 9.2 and 17.0 months, respectively. The use of PEMBRO, when compared with NIVO, had a significantly positive impact towards achieving an objective response, defined as either complete response or partial response (OR 5.54, *p* = 0.045). PFS and OS between the different TKIs or ICIs had no differences. The most adverse event was fatigue (36.4%), and most cases were mild and manageable. Conclusion: Combining TKI and ICI provides an acceptable antitumor efficacy in first-line therapy for advanced-stage HCC patients. The survival outcomes between different TKIs or ICIs display no differences.

## 1. Introduction

Liver cancer, mainly hepatocellular carcinoma (HCC), is the most common liver cancer, as well as a leading cause of cancer death globally [1]. For those with newly diagnosed HCC, many patients are presented with an intermediate (Barcelona Clinic of Liver Cancer stage B) or advanced stage (BCLC stage C) of the disease at their time of initial diagnosis [2]. Regarding the advanced stage (BCLC stage C) HCC, tyrosine kinase inhibitor (TKI) therapies play an essential role in the patient’s overall systematic therapy. Currently, approved first-line TKI therapies include sorafenib (SOR) and lenvatinib (LEN), according to meaningful phase 3 trials [3,4,5].

In the last few days, immune checkpoint inhibitor (ICI) therapies, including programmed cell death protein 1 (PD-1), programmed cell death protein ligand 1 (PD-L1), and cytotoxic T lymphocyte antigen 4 (CTLA-4), were approved for the treatment of several human cancers [6]. Currently, anti-PD-1 agents, including nivolumab (NIVO) or pembrolizumab (PEMBRO), are approved for treatment in unresectable HCC [7,8,9,10].

Lately, the IMbrave150 study, the combination of atezolizumab (anti-PD-L1 antibody) and bevacizumab (anti-VEGF monoclonal antibody), was accepted as a promising first-line treatment for advanced HCC [11]. These better results also suggest that there is a promising future for combining TKI and ICI therapies in the treatment of advanced HCC. However, the cost of atezolizumab and bevacizumab is high. Alternatively, another combination therapy involving TKIs, including SOR and LEN, plus ICIs, including NIVO and PEMBRO, have been better adapted in clinical practices.

The aim of the present study is to generate real-world data on patients with advanced-stage HCC that have received a combination of TKIs and ICIs as first-line therapy.

## 2. Methods

Data for patients with BCLC stage C HCC, and who were receiving a combination of TKIs and ICIs as first-line therapy at Taichung Veterans General Hospital from April 2019 to July 2021, were evaluated. HCC was diagnosed according to the American Association for the Study of Liver Disease (AASLD) guidelines [12]. All enrolled patients were categorized as Child-Pugh class A. The exclusion criteria included those patients diagnosed with Child-Pugh stage B or C, had an intolerance to combining therapy, a poor performance status (Eastern Cooperative Oncology Group performance score ≥ 2), a survival period of less than 2 months, and absence of a radiological images within the following day. The characteristics of the enrolled patients, including age, gender, presence of chronic hepatitis B (HBV), hepatitis C (HCV) infection, macroscopic vascular invasion (MVI), or extrahepatic spread (EHS), were all collected. Their laboratory data, including serum level of bilirubin, alanine aminotransferase (ALT), and alpha-fetoprotein (AFP), were also recorded for each individual.

After administering combination therapy, the patients received regular follow-up in the outpatient clinic every 2 weeks. The treatment of TKIs and ICIs usage was determined by each patient’s hepatologist. The mean dosage of TKIs and ICIs for each enrolled subject patient was also determined by each patient’s hepatologist according to each patient’s clinical condition.

Tumor response, as seen on radiological dynamic images, was assessed every 8 weeks by experienced radiologists. Treatment of TKI and ICI was discontinued once obvious tumor progression was disclosed through subsequent imaging studies. The mean dosage and therapeutic duration of TKIs and ICIs for each enrolled subject patient were subsequently recorded.

The best tumor response was assessed according to the modified RECIST (mRECIST) criteria [13], which included complete response (CR), partial response (PR), stable disease (SD), and progressive disease (PD). The patients showing either CR or PR were categorized into the objective response group. The objective response rate (ORR) and disease control rate (DCR) of patients’ tumors treated using combination therapy were calculated.

Adverse events were recorded as the appearance of hand-foot skin reaction (HFSR), hypertension, diarrhea, or fatigue after administration of combination therapy. The associations between different TKIs; ICIs; clinical parameters, including age, gender, serum level of AFP; presence of HBV, HCV, MVI, and EHS; and the efficacy of combination therapy were analyzed. Progression-free survival (PFS) was defined as the time from start of combination therapy administration to either radiological confirmation of tumor progression or death, and presented as median value with a 95% confidence interval (CI). Overall survival (OS) was defined as the time from start of combination therapy until death and also presented as a median value with a 95% CI.

Data are expressed as median and interquartile range (IQR) for each continuous variable and a percentage of the total patient number for each category variable. Statistical comparisons were made using Pearson’s chi-square test in order to compare category variable parameters. Mann–Whitney test was adapted to analyze continuous variable parameters. A *p*-value below 0.05 was defined as statistically significant. Survival analysis was applied using the Kaplan–Meier method for univariate and multivariate analysis and comparisons were subsequently performed with the log-rank test.

## 3. Results

### 3.1. Patient Characteristics

A total of 33 patients being treated with combination therapy were enrolled, with 8, 4, 11, and 10 cases receiving SOR/NIVO, SOR/PEMBRO, LEN/NIVO, and LEN/PEMBRO respectively. The general data of these patients are shown in Table 1. Overall, the median age was 66 years, with a male predominance (78.8%) being noted. The prevalence of chronic HBV and HCV infection was 57.6% and 24.2% respectively. All cases belonged to Child-Pugh class A and BCLC stage C. Sixteen (48.2%) and 19 patients (57.6%) had MVI and EHS, respectively. The median value of AFP was 130 ng/mL, with 13 patients (39.4%) having AFP ≥ 400 ng/mL at the baseline value prior to the administration of combination therapy. There were 10 patients (30.8%) who experienced an AFP decrease over 10% from the baseline value during the course of their combination therapy.

The mean oral daily doses of SOR and LEN were 366 mg and 7 mg respectively, while the mean intravenous doses given every 2 to 3 weeks of NIVO and PEMBRO were 120 mg and 158 mg, respectively. The mean usage duration periods of SOR, LEN, NIVO, and PEMBRO were 7.1, 7.8, 10.8, and 7.9 months, respectively.

### 3.2. Best Radiological Response

The outcomes for the enrolled patients are listed in Table 2. The numbers of patients with CR, PR, SD, and PD were 2 (6.1%), 14 (42.4%), 8 (24.2%), and 9 (27.3%), respectively. Overall, the ORR was 48.5%, and the DCR was 72.7%. Regarding the individuals treated with different combination therapy regimens, the patients receiving SOR/PEMBRO (75.0%) had the highest ORR, followed by LEN/PEMBRO (70.0%), LEN/NIVO (51.8%), and SOR/NIVO (25.0%).

Logistic analysis of the patients who achieved tumor objective response through combination therapy is shown in Table 3. Clinical parameters including age, gender, viral hepatitis; presence of MVI, EHS, and adverse effects; values of AFP; and different TKIs had non-significant effects. In contrast, the use of PEMBRO, when compared with NIVO, had a significantly positive impact (OR 5.42, 95% CI 1.19–24.52, *p* = 0.028), with the significance still existing after being adjusted through multivariable analysis (OR 5.54, 95% CI 1.06–28.91, *p* = 0.045).

### 3.3. Progression-Free Survival and Overall Survival

**Figure 1:**
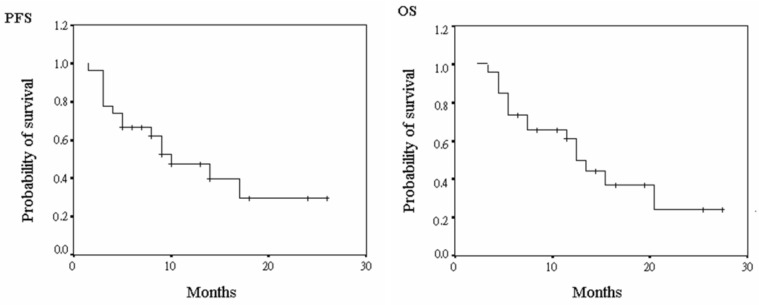
The survival time of hepatocellular carcinoma patients receiving combination therapy. (PFS, progress free survival; OS, overall survival).

**Figure 2:**
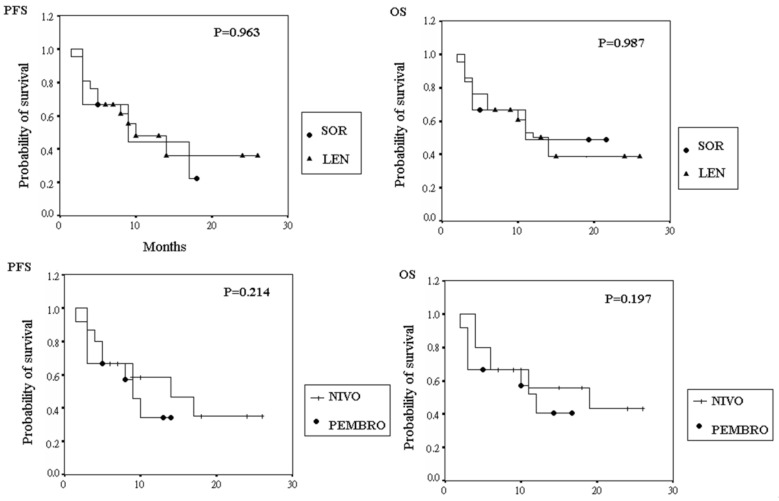
The survival time of hepatocellular carcinoma patients receiving different tyrosine kinase inhibitors and immune checkpoint inhibitors. (LEN, lenvatinib; NIVO, nivolumab; OS, overall survival; PEMBRO, pembrolizumab; PFS, progression free survival; SOR, sorafenib).

As shown in Figure 1, the PFS of all patients was 9.2 ± 6.4 months, and the OS 17.0 ± 6.3 months. Further analysis of both PFS and OS when stratified by different TKIs or ICIs is shown in Figure 2. PFS was 9.1 ± 6.8 months and 9.3 ± 6.4 months, and OS 17.0 ± 7.1 months and 17.0 ± 6.2 months, in patients with SOR and LEN, respectively. These differences were non-significant (*p* = 0.963 and 0.987). PFS was 9.6 ± 7.4 and 8.5 ± 4.4 months, and OS 17.4 ± 7.2 and 16.2 ± 4.6 months, in patients with NIVO and PEMBRO, respectively. Similarly, these differences were insignificant (*p* = 0.214 and 0.197).

### 3.4. Adverse Events

The adverse events detected in each group are shown in Table 4. Overall, the prevalences of HFRS, hypertension, diarrhea, and fatigue were 30.3%, 18.2%, 30.3%, and 36.4% respectively. All instances of hypertension occurred in the patients receiving LEN, while the cases receiving SOR had a higher prevalence of HFSR.

## 4. Discussion

HCC represents the most common type of cancer worldwide, with its incidence rate continuously increasing. However, although screening programs diagnose numerous HCCs at an early stage, still more than half of patients will be diagnosed at an advanced stage [2]. SOR is a TKI, targeting mainly VEGFR2, PDGFR, and KIT [14]. In the SHARP trial, SOR was associated with a significantly increased OS compared with patients receiving a placebo (10.7 vs. 7.9 months; *p* < 0.001). Additionally, PFS was shown to be significantly longer with SOR (5.5 vs. 2.8 months; *p* < 0.001) [3]. Similar efficacy was reported from the Asia-Pacific trial (OS: 6.5 vs. 4.2 months; HR: 0.68; *p* = 0.014) [4]. Therefore, when treating unresectable HCC, SOR has been used as the first systemic standard treatment since back in 2007.

LEN is another available TKI, targeting VEGFR1–3, FGFR1–4, PDGFR, RET, and KIT [15]. LEN was tested within the REFLECT trial, which acted as an open-label, multicenter, noninferiority trial comparing SOR with LEN in previously untreated patients. Although the study showed no significant improvements in median survival in the LEN arm (13.6 vs. 12.3 months), there was still a significant improvement seen in tumor response (ORR: 24.1 vs. 9.2%; *p* = 0.001) and time to progression (8.9 vs. 3.7 months; *p* < 0.0001) [5]. Currently, LEN has also been approved as a first-line treatment for patients with intermediate or advanced-stage HCC who have not received prior systemic therapy.

Unfortunately, regarding the current first-line TKIs for treating HCC, their disadvantages include a low ORR, easier to tumor progression, and a lack of long-term survival.

ICI therapy, particularly antibodies targeting the programmed cell death-1 (PD-1)/programmed cell death ligand-1 (PD-L1) pathway, represented a major breakthrough in drug development for oncologists during the past decade. Anti-PD-1 or anti-PD-L1 monotherapy has been approved for the treatment of more than 10 cancer types, with ORRs of 15–20% and good safety profiles being seen [6]. In the context of HCC, the CheckMate-040 study, a nonrandomized Phase I/II study that included 56 therapy-naive patients, suggested that NIVO may be meaningfully effective as first-line therapy for patients with advanced HCC (ORR 13%; with 6- and 9-month survival rates of 89 and 82%) [7]. However, the phase III randomized CheckMate-459 study involving a total of 743 patients who were receiving NIVO or SOR, disclosed no differences in either OS (16.4 vs. 14.7 months; *p* = 0.0752) or PFS (3.7 vs. 3.8 months), although tumor response was slightly better for NIVO (ORR: 15 vs. 7%) [10].

Similarly, for PEMBRO, the results of the Phase III randomized double-blind keynote-240 trial involving a total of 413 patients receiving either PEMBRO or a placebo, noted a non-significant OS (13.9 vs. 10.6 months; *p* = 0.0238, it did not meet the prespecified boundaries of *p* = 0.0174) and PFS periods (3.0 vs. 2.8 months; *p* = 0.0022, it did not meet the prespecified boundaries of *p* = 0.002) [9]. Based on the above studies, ICIs should not be used in the routine care of patients with untreated advanced HCC. However, the combining of TKIs and ICIs may be an alternative choice in the first-line treatment of advanced HCC.

Interstitial cells, including Kupffer cells; dendritic cells; liver endothelial cells; liver stellate cells; and immunosuppressive cytokines, including IL-10 or TGF-β, may play an important role in the development of the immunosuppressive environment of HCC. Combining TKI and IO is expected to improve this immunosuppressive microenvironment [16]. Regarding experimental models for HCC, SOR, in combination with NIVO, showed a stronger tumor growth inhibition as opposed to SOR or NIVO monotherapy [17]. In a phase 1b trial, LEN plus NIVO in patients with unresectable HCC revealed a high ORR of 76.7% and an ability to manage adverse events [18]. A phase 1b trial involving LEN plus PEMBRO as a first-line treatment for advanced HCC, reported an ORR as high as 36.0% (95% CI 26.6–46.2%) with a tolerable safety profile [19]. Furthermore, a phase 3 trial for the purpose of assessing the efficacy of the combination of LEN and PEMBRO as first-line treatment for HCC is now also underway.

This open-label phase 3 IMbrave150 trial compared the combination of atezolizumab and bevacizumab with SOR in untreated patients diagnosed with advanced unresectable HCC. The ORR between the two approaches was 27.3% vs. 11.9%, with a 5.5% complete response seen in the atezolizumab and bevacizumab group. The PFS was 6.8 months (95% CI 5.7–8.3) for the atezolizumab/bevacizumab group, with the HR for mortality being 0.58 (95% CI 0.42–0.79; *p* = 0.001) in favor of atezolizumab/bevacizumab [11].

For our 33 patients receiving combination therapy as first-line treatment for advanced stage HCC, the ORR and DCR were 48.5% and 72.7%, respectively, with the average PFS and OS being 9.2 and 17.0 months, respectively. Radiological CR occurred in two patients (6.1%). The antitumor results of our study were shown to be better than previous trials involving monotherapy with TKI or ICI, and similar to studies using combination regimens. The average dose of TKI (SOR 366 mg and LEN 7 mg per day) and ICI (NIVO 120 mg and PEMBRO 158 mg every 2–3 weeks) administered to our patients was low, with the reasons for that possibly being concern over drug-associated adverse effects and economic factors, as ICI treatment is not currently covered by national health insurance in Taiwan. Certainly, the low doses of TKI and ICI may cause a negative impact on treatment efficacy for HCC.

Further analysis of our data determined that achieving an objective response was significantly associated with different ICIs. The prescription of PEMBRO, when compared with NIVO, had a significantly positive impact towards achieving tumor objective response (OR 5.54, *p* = 0.045). This may be due to a relatively acceptable average dose of PEMBRO (158 mg every 3 weeks) when compared with NIVO (120 mg every 2 weeks). The patients receiving NIVO and those receiving PEMBRO showed no differences in PFS (9.6 vs. 8.5 months; *p* = 0.214) or OS (17.4 vs. 16.2 months; *p* = 0.197). As for the different TKIs, the antitumor responses were similar both in the radiological responses and survival time.

The most adverse events our patients experienced were fatigue (36.4%), followed by HFSR (30.3%), diarrhea (30.3%) and hypertension (18.2%). Not surprisingly, HFRS usually occurred in the patients receiving SOR, with all hypertension happening in the patients receiving LEN. The incidences of adverse events in our patients are similar with previous trials, with most being mild and manageable. However, the rate of adverse events in our study may be underestimated due to its retrospective design. Additionally, our enrolled patients followed good compliance to combination therapy, so because certain individuals who experienced severe adverse events were excluded, their data may have been excluded. Besides, some data about immune-related adverse events (IRAEs), such as pneumonitis, thyroiditis, or hepatitis, may be missed in our study.

There were several limitations to our study. First, this study was designed as being retrospective and was conducted at a single tertiary care center. Selection bias may therefore have existed. Second, only subjects diagnosed with Child–Pugh class A and BCLC stage C HCC were enrolled in our study. Third, our sample size was small and the follow-up period was short. Four, alcohol consumption or other underlying diseases, such as immune diseases and non-alcoholic fatty liver disease (NAFLD), may influence the therapeutic response of the enrolled patients, and these data were unavailable in our study. Lastly, no sufficient data surrounding the regimen of atezolizumab/bevacizumab was obtained and compared. Further prospective research involving the analysis of more variables is therefore warranted.

## 5. Conclusions

Combining TKIs and ICIs provides an acceptable antitumor efficacy in first-line therapy for advanced-stage HCC. The use of PEMBRO, as compared with NIVO, makes it easier to achieve a tumor-objective response. The survival outcomes seen between either different TKIs or ICIs show no differences.

## Figures and Tables

**Table 1:** General characteristics of patients.

		All (N = 33)				SOR/NIVO (N = 8)				SOR/PEMBRO (N = 4)				LEN/NIVO (N = 11)				LEN/PEMBRO (N = 10)				*p*-Value
		M	(IQR)	N	%	M	(IQR)	N	%	M	(IQR)	N	%	M	(IQR)	N	%	M	(IQR)	N	%	
Age (years)		66	(14)			68	(9)			70	(4)			60	(18)			56	(15)			0.090 ^a^
Gender (male)				26	(78.8%)			6	(75.0%)			2	(50.0%)			8	(72.7%)			10	(100%)	0.173 ^b^
Hepatitis infection	HBV			19	(57.6%)			4	(50.0%)			0				8	(72.7%)			7	(70.0%)	0.063 ^b^
	HCV			8	(24.2%)			0				4	(100%)			2	(18.2%)			2	(25.0%)	0.062 ^b^
Child-Pugh stage	A			33	(100%)			8	(100%)			4	(100%)			11	(100%)			10	(100%)	1.000 ^b^
BCLC stage	C			33	(100%)			8	(100%)			4	(100%)			11	(100%)			10	(100%)	1.000 ^b^
MVI				16	(48.2%)			4	(50.0%)							6	(54.5%)			6	(60.0%)	0.215 ^b^
EHS				19	(57.6%)			4	(50.0%)			4	(100%)			6	(54.5%)			5	(50.0%)	0.332 ^b^
Bilirubin (U/L)		0.7	(0.9)			1.4	(1.9)			0.5	(0.2)			0.7	(0.5)			0.9	(0.8)			0.281 ^a^
ALT (U/L)		36	(34)			34	(25)			15	(15)			51	(45)			40	(19)			0.206 ^a^
Abnormal ALT (male > 50 U/L or female > 35 U/L)				10	(30.3%)			2	(25.0%)			0				6	(54.5%)			2	(20.0%)	0.144 ^b^
AFP (ng/mL)		130	(3115)			227	(3408)			1036	(2067)			132	(3916)			34	(7502)			0.575 ^a^
Abnormal AFP (AFP > 7 ng/mL)				22	(66.7%)			6	(75.0%)			2	(50.0%)			9	(81.8%)			5	(50.0%)	0.371 ^b^
AFP (ng/mL)	≥400			13	(39.4%)			2	(25.0%)			2	(50.0%)			5	(45.5%)			4	(40.0%)	0.788 ^b^
	<400			20	(60.6%)			6	(75.0%)			2	(50.0%)			6	(54.5%)			6	(60.0%)	
AFP decreased > 10%				10	(30.3%)			4	(50.0%)			0				4	(36.4%)			2	(20.0%)	0.272 ^b^

All *p*-values were analyzed with Mann–Whitney test ^a^ and Pearson’s Chi-square test ^b^. Abbreviations: AFP, alpha-fetoprotein; ALT, alanine aminotransferase; EHS, extrahepatic spread; HBV, Hepatitis B; HCV, Hepatitis C; IQR, interquartile range; LEN, lenvatinib; M, median; MVI, macroscopic vascular invasion; N, number of patients; NIVO, nivolumab; PEMBRO, pembrolizumab; SOR, sorafenib.

**Table 2:** Best radiological responses.

		All (N = 33)		SOR/NIVO (N = 8)		SOR/PEMBRO (N = 4)		LEN/NIVO (N = 11)		LEN/PEMBRO (N = 10)		*p*-Value
		N	%	N	%	N	%	N	%	N	%	
mRECIST												0.279
	CR	2	(6.1%)	0		1	(25.0%)	1	(7.4%)	0		
	PR	14	(42.4%)	2	(25.0%)	2	(50.0%)	3	(44.4%)	7	(70.0%)	
	SD	8	(24.2%)	2	(25.0%)	1	(25.0%)	4	(22.3%)	1	(10.0%)	
	PD	9	(27.3%)	4	(50.0%)	0		3	(25.9%)	2	(20.0%)	
ORR		16	(48.5%)	2	(25.0%)	3	(75.0%)	4	(51.8%)	7	(70.0%)	0.145
DCR		24	(72.7%)	4	(50.0%)	4	(100%)	8	(74.1%)	8	(80.0%)	0.278

All *p*-values were analyzed with Pearson’s Chi-square test. Abbreviations: CR, complete response; DCR, disease control rate; LEN, lenvatinib; N, number of patients; NIVO, nivolumab; ORR, objective response rate; PD, progressive disease; PEMBRO, pembrolizumab; PR, partial response; SD, stable disease; SOR, sorafenib.

**Table 3:** Logistic analysis of each item to achieve objective response.

	Univariate Analysis			Multivariate Analysis		
	HR	(95% CI)	*p*-Value	HR	(95% CI)	*p*-Value
Age (≤65 vs. >65 years)	1.87	(0.44–7.85)	0.395	1.07	(0.13–8.96)	0.948
Gender (male vs. female)	8.17	(0.85–77.97)	0.068	8.69	(0.66–114.26)	0.100
HBV (HBsAg + vs. −)	0.90	(0.23–3.58)	0.881			
HCV (anti-HCV + vs. −)	4.50	(0.75–26.93)	0.099			
AFP (≤400 vs. >400 ng/mL)	1.96	(0.47–8.11)	0.356			
AFP decreased > 10% (yes vs. no)	1.64	(0.36–7.38)	0.521			
MVI (yes vs. no)	0.42	(0.10–1.70)	0.224			
EHS (yes vs. no)	0.55	(0.14–2.20)	0.394			
TKI (LEN vs. SOR)	1.54	(0.37–6.45)	0.554	1.27	(0.15–11.09)	0.826
ICI (PEMBRO vs. NIVO)	5.42	(1.19–24.52)	0.028	5.54	(1.06–28.91)	0.042
HFRS (yes vs. no)	1.95	(0.43–8.82)	0.386			
Hypertension (yes vs. no)	1.29	(0.10–3.27)	0.849			
Diarrhea (yes vs. no)	1.09	(0.25–4.81)	0.908			
Fatigue (yes vs. no )	3.00	(0.92–23.45)	0.057			

Abbreviations: AFP, alpha-fetoprotein; CI, confidence interval; EHS, extrahepatic spread; HBV, Hepatitis B; HCV, Hepatitis C; HR, hazard ratio; ICI, immune checkpoint inhibitor; LEN, lenvatinib; MVI, microscopic vascular invasion; N, number of patients; NIVO, nivolumab; PEMBRO, pembrolizumab; SOR, sorafenib; TKI, tyrosine kinase inhibitor.

**Table 4:** Adverse events of combination therapy.

	All (N = 33)		SOR/NIVO (N = 8)		SOR/PEMBRO (N = 4)		LEN/NIVO (N = 11)		LEN/PEMBRO (N = 10)		*p*-Value
	N	%	N	%	N	%	N	%	N	%	
HFRS	10	(30.3%)	4	(50.0%)	2	(50.0%)	1	(16.7%)	3	(30.0%)	0.208
Hypertension	6	(18.2%)	0		0		2	(18.2%)	4	(40.0%)	0.118
Diarrhea	10	(30.3%)	2	(25.0%)	2	(50.0%)	3	(27.3%)	3	(30.0%)	0.828
Fatigue	12	(36.4%)	2	(25.0%)	2	(50.0%)	5	(45.5%)	3	(30.0%)	0.721

All *p*-values were analyzed with Pearson’s Chi-square test. Abbreviations: HFRS, hand foot syndrome reaction; LEN, lenvatinib; N, number of patients. NIVO, nivolumab; PEMBRO, pembrolizumab; SOR, sorafenib.

## Data Availability

Not applicable.
